# Structural and Functional Deficits in Patients with Poststroke Dementia: A Multimodal MRI Study

**DOI:** 10.1155/2021/3536234

**Published:** 2021-11-03

**Authors:** Huaying Cai, Zhiyong Zhao, Linhui Ni, Guocan Han, Xingyue Hu, Dan Wu, Xianjun Ding, Jin Wang

**Affiliations:** ^1^Department of Neurology, Neuroscience Center, Sir Run Run Shaw Hospital, Zhejiang University, Hangzhou, China; ^2^Key Laboratory for Biomedical Engineering of Ministry of Education, Department of Biomedical Engineering, College of Biomedical Engineering & Instrument Science, Zhejiang University, Hangzhou, China; ^3^Department of Radiology, Sir Run Run Shaw Hospital, Zhejiang University, Hangzhou, China; ^4^Department of Orthopaedic Surgery, Sir Run Run Shaw Hospital, Zhejiang University, Hangzhou, China

## Abstract

Although many neuroimaging studies have reported structural and functional abnormalities in the brains of patients with cognitive impairments following stroke, little is known about the pattern of such brain reorganization in poststroke dementia (PSD). The present study was aimed at investigating alterations in spontaneous brain activity and gray matter volume (GMV) in PSD patients. We collected T1-weighted and resting-state functional magnetic resonance imaging data from 20 PSD patients, 24 poststroke nondementia (PSND) patients, and 21 well-matched normal controls (NCs). We compared the differences among the groups in GMV and the fractional amplitude of low-frequency fluctuations (fALFF). Then, we evaluated the relationship between these brain measures and cognitive assessments and explored the possible distinguisher for PSD by receiver operating characteristic (ROC) curve analysis. PSD patients showed smaller GMV in the right superior temporal gyrus and lower fALFF values in the right inferior frontal gyrus than both PSND patients and NCs, but such differences were not observed between PSND patients and NCs. Moreover, GMV in the left medial prefrontal cortex showed a significant positive correlation with the Mini-Cog assessment in PSD patients, and GMV in the left CPL displayed the highest area under the ROC curve among all the features for classifying PSD versus PSND patients. Our findings suggest that PSD patients show dementia-specific structural and functional alteration patterns, which may help elucidate the pathophysiological mechanisms underlying PSD.

## 1. Introduction

Poststroke dementia (PSD), irrespective of the presumed cause, is a clinical entity that encompasses all types of dementia following a stroke and characterized as cognitive decline [1]. Previous studies have reported the prevalence of poststroke dementia (PSD) ranging from 13% to 27% [2–4], and this variation may be related to many factors, including race [5, 6], educational level [7], economic level [4], lifestyle [8], and aging population [3]. Moreover, PSD may have potential influences on various aspects of daily living activities, especially stroke recurrence [9] and functional outcome [10], and has become a significant public health burden [11]. Although previous neuroimaging studies based on structural or functional MRI have attempted to explore PSD-related patterns in brain reorganization [12–14, 42], few studies used multimodal MRI to explore the neural mechanisms underlying PSD.

In the neuroimaging field, functional magnetic resonance imaging (fMRI) has been widely used to investigate the pathogenesis of neurological diseases [15–17]. Compared with task-based fMRI, resting-state fMRI (rs-fMRI) shows the advantage of application in stroke studies since it does not require specific tasks [18, 19]. rs-fMRI mainly examines the low-frequency (0.01-0.08 Hz) fluctuations in blood oxygenation level-dependent (BOLD) fMRI signals at rest [20, 21]. As a valid method to detect local spontaneous neuronal activity [22], the amplitude of low-frequency fluctuations (ALFF) has been used to study various cognitive and neuropsychiatric disorders, including mild cognitive impairment [23] and schizophrenia [24]. However, compared with ALFF, the fractional amplitude of low-frequency fluctuations (fALFF) could be robust against nonspecific signal components [25], allows the analysis of frequency-specific activity [26], and improves the sensitivity and specificity in detecting regional spontaneous brain activity [27]. Using this method, previous stroke studies found that compared to normal controls (NCs), patients with acute cerebellar infarction showed increased fALFF values in the right frontal lobe, left hippocampus, and right cingulate gyrus and decreased fALFF values in the cerebellum posterior lobe (CPL) [18]; stroke patients with depression symptoms have higher fALFF values in the left dorsolateral prefrontal cortex and the right precentral gyrus compared to nondepressed patients [26]. Moreover, depressive symptom scores in stroke patients were positively correlated with fALFF values in the left insula, superior temporal lobe, thalamus, cerebellum, and right caudate [28]. These findings suggest that fALFF could be used to explore neuronal functional alterations in patients after stroke. However, this method has never been used to detect changes in spontaneous neural activity in PSD patients.

In addition, gray matter volume (GMV), quantitatively calculated by voxel-based morphometry (VBM) [29] analysis of T1-weighted images, has become an ideal morphological measurement to explore structural alterations in poststroke patients [30, 31]. For example, Stebbins et al. found that patients with cognitive impairments after stroke showed significant GMV reductions in the thalamus, cingulate gyrus, and frontal, temporal, parietal, and occipital lobes compared with patients without cognitive impairments [32]. Ahn et al. observed significantly lower GMV in the bilateral cerebellum in chronic stroke patients with cognitive impairments than in NCs [33]. In addition, Yang and colleagues reported that compared with NCs, patients with poststroke aphasia showed increased GMV in the right superior temporal gyrus, right inferior parietal lobule, and left middle occipital gyrus and decreased GMV in the right caudate gyrus and bilateral thalami [34]. These results indicated that patients with different poststroke cognitive dysfunctions show different GMV alteration patterns. However, the GMV alterations in PSD patients remain unclear, and the utility of structural neuroimaging studies with MRI has not been fully explored.

The current study was aimed at exploring the potential structural and functional changes using fALFF and GMV methods in patients with PSD. Based on previous findings of alterations in fALFF and GMV in patients with different poststroke dysfunctions [18, 26, 32], we hypothesized that disease-specific patterns of fALFF and GMV changes would be discovered in patients with PSD. Furthermore, based on the report that clinical symptoms were correlated with neural alterations in stroke patients [23], we also hypothesized that the fALFF/GMV alterations in patients with PSD would be related to their cognitive functions.

## 2. Materials and Methods

### 2.1. Participants

Fifty-nine poststroke patients (PSD/PSND: *N* = 25/34) and twenty-five NCs were recruited at Sir Run Run Shaw Hospital from September 2017 to October 2019. The Institutional Review Board provided ethical approval of this study at the local hospital. All participants provided informed consent. The inclusion criteria for the patients were as follows: (1) age > 18 years, (2) complement of neuropsychological tests at the acute phase and the third month after stroke, (3) eligibility for a scan between 1 and 6 months after stroke onset, (4) first-episode stroke, and (5) definitive acute ischemic stroke based on DWI of the head. The exclusion criteria for all participants were as follows: (1) any neuropsychiatric comorbidity such as depression (total score ≥ 8 on the17-item Hamilton Depression Rating Scale), anxiety (total score ≥ 7 on the Hamilton Anxiety Rating Scale), epilepsy, brain tumor, brain trauma, and drug or alcohol abuse; (2) any clinically significant or unstable medical disorder; (3) any contraindication for MRI; (4) prestroke dementia (-Informant Questionnaire on Cognitive Decline in the Elderly (IQCODE) score > 3:31); and (5) aphasia before or after the stroke. A total of 19 subjects were excluded because of the excessive head motion (>2 mm/degree) (4 PSD patients, 7 PSND patients, and 4 NCs) and lesion volume (>10 ml) (1 PSD and 3 PSND patients). Finally, based on matching age and education among the three groups, 21 NCs, 20 PSD, and 24 PSND patients were included in the final analysis in the present study.

### 2.2. Clinical Assessments

The National Institutes of Health Stroke Scale (NIHSS) and modified Rankin Scale (mRS) scores were recorded upon patient consent. According to the poststroke cognitive impairment assessment guideline by the Chinese Stroke Centre Alliance, the IQCODE was used to evaluate the prestroke cognitive status of each patient [35], and both the Mini-Mental State Examination (MMSE) [36] and Mini-Cog [37] assessments were used to evaluate the cognitive performance of each patient both at the acute stage and at the third month poststroke. We controlled for vascular risk factors, such as blood pressure, blood lipids, blood sugar, and smoking, for each patient after stroke via antihypertensive drugs, glucose-lowering drugs, and lipid-lowering drugs. All patients underwent standardized treatment based on the “Guideline for Early Management of Adults with Ischemic Stroke” [38] and were followed up in the outpatient clinic with the same clinician. All normal controls underwent cognitive assessment (both the MMSE and Mini-Cog) before the MRI scan. Two neurologists (HY C and LH N) who were blinded to the MRI data recorded the clinical data and performed the cognitive examinations.

PSD was diagnosed by two neurologists with 15 years of experience and 4 years of experience according to the 2019 Chinese Vascular Cognitive Impairment Guideline, which defines PSD as a status in which cognitive impairment lasts for three months after a stroke [39]. We therefore determined the diagnosis at three months after stroke onset, which is also consistent with the international consensus [40] (within 6 months). PSD was identified if the patient satisfied one of the following two criteria: (1) MMSE scores below a certain cutoff value depending on the education level: (i) MMSE < 24 for patients with education higher than junior middle school, (ii) MMSE ≤ 19 for patients with primary school education, or (iii) MMSE ≤ 17 for patients with illiteracy, and (2) an adjusted Mini-Cog score of <3. Finally, poststroke patients were divided into two subgroups (PSD and PSND).

### 2.3. MRI Data Acquisition

Multimodal MRI scans, including resting-state fMRI and T1- and diffusion-weighted images, were performed for each patient approximately three months after stroke. All data were acquired on a Siemens 3 T MAGNETOM Skyra MRI scanner (Siemens Healthcare, Erlangen, Germany) with a 20-channel head coil. The sequences and parameters were identical to those in our previous study [41].

### 2.4. Lesion Analysis

**Figure 1:**
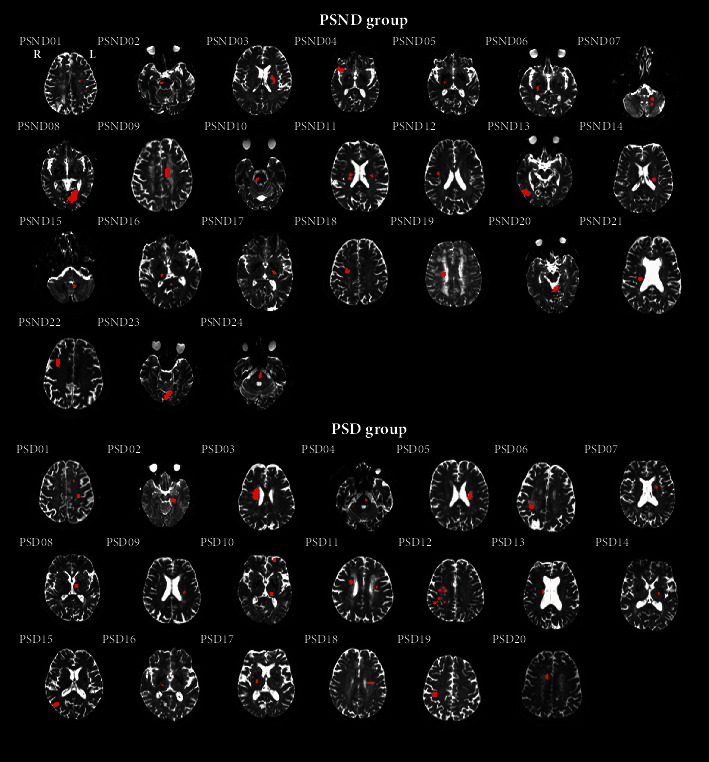
Lesion display for each patient in the PSD and PSND groups. The red region represents an individual lesion.

We manually drew lesion regions slice by slice on the nondiffusion-weighted image (b0) using MRIcro software (http://www.mricro.com) (Figure 1), and lesion masks were confirmed by two neurologists (HY C and LH N). Then, we determined the location and number of lesions and calculated the lesion volume for each patient.

### 2.5. FMRI Data Processing

We preprocessed the resting-state fMRI data using the *Advanced* DPARSF (http://www.restfmri.net) and SPM12 (http://www.fil.ion.ucl.ac.uk/spm) toolkits. The first 5 functional volumes were discarded, and the remaining 115 volumes underwent slice timing and head motion corrections. Then, white matter, cerebrospinal fluid, and the Friston 24-parameter model of head motion were regressed out as nuisance variables. Next, the data were spatially normalized to an EPI template in the MNI space. Finally, we conducted the fALFF analysis. Specifically, a ratio of the low-frequency amplitude within 0.01-0.1 Hz from fast Fourier transformation to the power spectrum of the entire frequency range was computed at each voxel to obtain fALFF values [25]. The fALFF maps were normalized by subtracting the mean value for the entire brain and then dividing by the whole-brain standard deviation. The maps were further smoothed by a Gaussian kernel at a full width half maximum (FWHM) of 6 mm [42, 43].

### 2.6. VBM Analysis

We performed the VBM analysis with SPM12. We first registered the T1 images to the Montreal Neurological Institute (MNI) template and then segmented the whole-brain structural data into white matter, gray matter, and cerebrospinal fluid. We conducted bias correction to remove intensity nonuniformities. Segmented images of the gray matter were preserved to assess the number of volume changes based on spatial registration, and the modulated images of the gray matter could reflect the tissue volumes for using VBM analysis. Finally, we smoothed the normalized gray matter images using an 8 mm FWHM Gaussian filter [42, 43].

### 2.7. Statistical Analysis

We first performed one-way ANCOVA to compare fALFF and GMV maps among the three groups within a gray matter mask with age, sex, education, head motion, intracranial volume (ICV) and volume, location, and number of lesions as the covariates. A two-tailed Gaussian random field correction with a voxel-level *p* < 0.01 and a cluster-level *p* < 0.05 was used to control false discoveries due to multiple comparisons. Then, for the regions showing a significant group-level main effect, post hoc *t*-tests were performed to detect the pairwise differences in fALFF and GMV (Bonferroni corrected, *p* < 0.05). Next, to examine the relationships between the fALFF/GMV values in the regions with between-group differences and cognitive functions (MMSE or Mini-Cog scores) and between fALFF and GMV values, partial correlation analyses were separately performed in each patient group while controlling for age, sex, education, head motion, ICV and volume, location, and number of lesions. Finally, we used the fALFF/GMV in the regions showing significant differences between groups as the feature to perform receiver operating characteristic (ROC) curve analysis to discriminate PSD from PSND patients.

## 3. Results

The three groups did not have significant differences in age, handedness, head motion, or intracranial volume (Table 1). Additionally, the duration of illness and stroke severity were matched between the two patient groups. Notably, sex and education showed significant differences between NCs and the patient groups but not between PSD and PSND (sex: *p* = 0.053; education: *p* = 0.60). Cognition scores (MMSE and Mini-Cog) in the PSD patients were significantly lower than those in the PSND group (Table 1). In addition, the two patient subgroups did not have significant differences in the volume, location, or number of stroke lesions (Table 2). None of the patients showed hemorrhagic transformation after stroke. The number of patients with cortical/subcortical lesions was 4/20 in the PSND group and 4/16 in the PSD group (Table 3).

**Figure 2:**
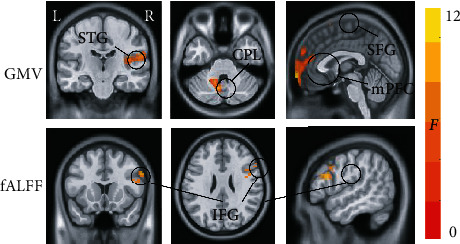
The regions showing significant differences in GMV/fALFF values among the PSND, PSD, and NC groups. GMV: gray matter volume; fALFF: fractional amplitude of low-frequency fluctuation; STG: superior temporal gyrus; CPL: cerebellum posterior lobe; SFG: superior frontal gyrus; mPFC: medial prefrontal cortex; IFG: inferior frontal gyrus; L: left; R: right. Color bar represents *F* values.

**Figure 3:**
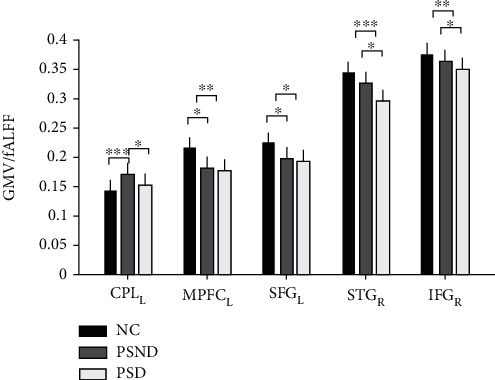
The differences in GMV and fALFF values among the PSND, PSD, and NC groups. GMV: gray matter volume; fALFF: fractional amplitude of low-frequency fluctuation; CPL_L: left cerebellum posterior lobe; mPFC_L: left medial prefrontal cortex; SFG_L: left superior frontal gyrus; STG_R: right superior temporal gyrus; IFG_R: right inferior frontal gyrus. ∗, ∗∗, and ∗∗∗ represent *p* < 0.05, *p* < 0.01, and *p* < 0.001, respectively.

ANCOVA found that GMV showed significant differences between the three groups in the left CPL, left medial prefrontal cortex (mPFC), superior frontal gyrus (SFG), and right superior temporal gyrus (STG); significant differences in fALFF values were observed in the right inferior frontal gyrus (IFG) (Figure 2 and Table 4). Post hoc analysis showed that both PSND and PSD patients had smaller GMV than NCs in the left medial prefrontal cortex (mPFC) and SFG; the left CPL displayed larger GMV in the PSND group compared with the NC and PSD groups. Importantly, we found dementia-specific changes in which the PSD group showed decreased GMV in the right STG and decreased fALFF in the right IFG compared with PSND and NC groups, but such differences were not found between PSND and NC groups (Figure 3).

**Figure 4:**
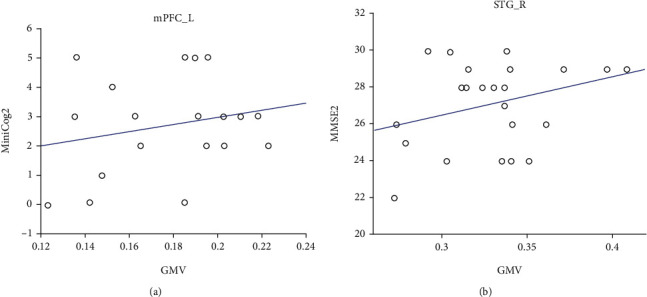
Correlations between clinical cognitive assessments and GMV in the mPFC_L (a) and STG_R (b) in different groups. GMV: gray matter volume; miniCog2: Mini-Cog assessment at the third month; MMSE2: Mini-Mental State Examination assessment at the third month; mPFC_L: left medial prefrontal cortex; STG_R: right superior temporal gyrus.

**Figure 5:**
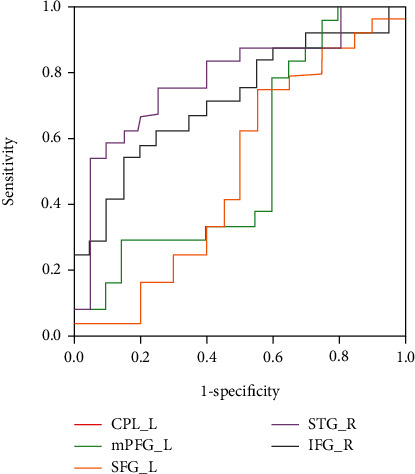
The receiver operating characteristic (ROC) curves for using GMV/fALFF values for the classification of PSND versus PSD patients. Specifically, the AUC in the left cerebellum posterior lobe, left medial prefrontal cortex, left superior frontal gyrus, right superior temporal gyrus, and right inferior frontal gyrus are 0.804, 0.531, 0.502, 0.783, and 0.717, respectively. AUC: area under the curve; fALFF: fractional amplitude of low-frequency fluctuation; CPL_L: left cerebellum posterior lobe; mPFC_L: left medial prefrontal cortex; SFG_L: left superior frontal gyrus; STG_R: right superior temporal gyrus; IFG_R: right inferior frontal gyrus.

Moreover, we found significant positive correlations between GMV in the left mPFC and Mini-Cog scores at the third month in the PSD group (*p* = 0.04, *r* = 0.56) and between GMV in the right STG and MMSE scores at the third month in the PSND group (*p* = 0.03, *r* = 0.52) (Figure 4), although they did not pass the Bonferroni correction of *p* < 0.05. In addition, ROC analysis showed that GMV in the left CPL and right STG and fALFF values in the right IFG significantly discriminated the PSD patients from the PSND patients (Figure 5 and Table 5). Specifically, the AUC in left CPL, left mPFC, left SFG, right STG, and right IFG were 0.804, 0.531, 0.502, 0.783, and 0.717, respectively. Also, the performance was improved (AUC = 0.898, *p* < 0.001) after combining the three brain regions with high AUC values5.

## 4. Discussion

The present study evaluated alterations in GMV and fALFF in patients with PSD, and the results supported our hypotheses that (1) PSD patients showed dementia-specific decreases in GMV in the right STG and fALFF in the right IFG; (2) GMV in the left mPFC in the PSD group was significantly positively correlated with Mini-Cog scores at the third month, and such a relationship was also found between GMV in the right STG in the PSND group and MMSE scores at the third month; and (3) fALFF values in the right IFG and GMV in the left CPL and right STG may be used to discriminate PSD patients from PSND patients. These findings provide a new insight into the neurophysiological mechanisms underlying PSD, which may motivate the development of a theoretical basis for clinical diagnosis.

### 4.1. PSD-Related Structural Alterations

As two core components of the prefrontal cortex, the medial gyrus and superior frontal gyrus have been linked to a variety of cognitive functional domains, especially in memory [44] and cognitive control [45]. Compared with NCs, Bhalsing et al. found GMV loss in the right mPFC in essential tremor patients with cognitive impairments [46]; Yang et al. reported significantly reduced GMV in the left SFG in silent cerebral infarction patients with cognitive impairment [47]; Li et al. observed a GMV reduction in the SFG in subcortical vascular dementia patients [48]. Moreover, Stebbins and colleagues reported decreased GMV in the bilateral mPFC and SFG in stroke patients with impaired cognitive performance compared with those without cognitive impairment [32]. These studies collectively demonstrated decreased GMV in the SFG and mPFC in patients with cognitive dysfunction, and the present study provided supporting evidence that PSD patients displayed GMV reductions in these two regions compared with NCs. Additionally, previous neuroimaging studies revealed GMV atrophy in the mPFC and SFG areas in transient ischemic attack patients [49], poststroke pain patients [50], and poststroke dysphagia patients [51]. Notably, the stroke patients in these studies did not have cognitive dysfunction, implying that GMV decreases in these two regions may be related to impairments in attentional and executive control in stroke patients [52, 53]. Consistently, the present study also found that PSND patients had a smaller GMV than NCs in the left mPFC and SFG. Hence, we speculate that reduced GMV in the left mPFC and SFG are correlated with both stroke and dementia, which may help to further understand the neural mechanisms underlying PSD.

Furthermore, the cerebellum not only is involved in motor function [54, 55] but also acts as a general modulator due to the presence of cerebellar activations in higher cognitive functions [56]. Previous studies have found significant reductions in GMV in the right cerebellar region in patients with subcortical vascular dementia [57] and in left cerebellar subfields in remitted major depression patients with persistent cognitive deficits [58]. Similar results were also observed in poststroke patients [33, 59]. However, one study revealed that patients with left hemisphere subcortical stroke showed increased GMV in the ipsilesional cerebellum VI [60]. These findings suggest that the decreased GMV in the cerebellum-related region in PSD compared with PSND in the present study may be associated with both cognitive dysfunction and stroke. Moreover, the increased GMV in the cerebellum in PSND patients compared with NCs may represent motor compensation after stroke [61].

### 4.2. Dementia-Specific Structural and Functional Alterations

The superior temporal gyrus is considered a key structure involved in cognitive processing [62, 63]. Previous neuroimaging studies have explored structural alterations in the STG in patients with dementia or cognitive impairments. For instance, one study based on VBM analysis showed that Parkinson's disease patients with dementia had a significant decrease in GMV in the bilateral STG compared to those without dementia [64]. Using a similar method, another study revealed that compared with NCs, patients with subcortical vascular mild cognitive impairment exhibited atrophy in the bilateral STG [48]. These findings suggest that the STG might be a potential neural biomarker in cognitive impairment diseases. Consistent with previous studies, the present study found that the right STG displayed a smaller GMV in PSD patients than in PSND patients and NCs, but such a difference was not observed between PSND patients and NCs, which implied that the structural reduction in the right STG was more likely to be specific to dementia rather than stroke. Moreover, studies have demonstrated that functional alterations in the inferior frontal gyrus (IFG), which is also considered to be related to cognition [65], have been reported in several previous neuroimaging studies. For example, Zhong et al. reported decreased ALFF in the left opercular part of the IFG in patients with cognitive control impairment compared with NCs [66]. Han et al. and Li et al. both observed that compared with NCs, patients with mild cognitive impairment had decreased ALFF values in the left IFG [67, 68]. Agreeing with these studies, the present study demonstrated decreased fALFF values in the right IFG in the PSD patients compared with the PSND patients and NCs, whereas the PSND patients did not show significant GMV changes in this region compared with the NCs. This suggests that the functional alterations in the right IFG could be a related biomarker of dementia in PSD patients. Inspired by all the findings, we speculate that exploring the specific structural and functional alterations in the right STG and IFG might facilitate a deeper understanding of the pathological mechanisms underlying PSD.

### 4.3. Clinical Implications of the Structural Alterations

Several studies have focused on the relationships between structural alterations in the mPFC/STG and cognitive function. For example, Bhalsing et al. demonstrated a positive correlation between GMV in the right mPFC and visual memory in patients with cognitive impairments [39]. Vidoni and colleagues reported that poor performance on cognitive measures was associated with lower GMV in the mPFC in subjects with early-stage AD [69]. Tong et al. found a significant positive correlation between cognitive maturity and GMV in the STG in young normal participants with relatively low cognitive maturity [70]. In addition, Ren et al. showed significant positive correlations between gray matter density in the left STG and accuracy of object working memory in healthy college students [71]. Similarly, the present study found significant positive correlations between GMV in the left mPFC and Mini-Cog scores at the third month in the PSD group and between GMV in the right STG and MMSE scores at the third month in the PSND group. Hence, we speculate that GMV reductions in the left mPFC and right STG may serve as a biomarker to predict cognitive function in poststroke patients. Meanwhile, using ROC analysis, the present study indicated that GMV in the left CPL has the best performance to discriminate PSD patients from PSND patients among all features. Consistently, previous studies found that structural alterations in cerebellar regions could distinguish dementia with Lewy bodies and Alzheimer's disease (AD) [72] and could discriminate AD patients from non-AD patients [73]. Therefore, decreased GMV in the CPL may be a prospective indicator to identify dementia among patients after stroke.

### 4.4. Limitations

Several limitations in the present study should be noted. First, the sample size was relatively small, and a larger sample size would be necessary to confirm our findings. Second, the stroke patients recruited in the present study included cortical and subcortical lesions in the bilateral hemispheres. Although we controlled for the lesions in the statistical analysis, they may still have had a certain impact on the results. In the future, we need to explore the alterations in GMV and fALFF values in PSD patients with unilateral lesions. Third, the current study was cross-sectional; therefore, we were unable to capture dynamic abnormalities in brain structure and function in PSD patients. A longitudinal study in the future may be effective in resolving this problem.

## 5. Conclusion

In summary, the present study was the first to use multimodal MRI data to detect alterations in GMV and fALFF in patients with PSD and found that PSD patients showed dementia-related GMV reductions in the left CPL and right STG and a decrease in fALFF in the right IFG. Moreover, GMV in the left mPFC showed a significant positive correlation with Mini-Cog scores in PSD patients, and GMV in the left CPL could effectively distinguish PSD from PSND patients. Taken together, these findings could provide new evidence to understand the neurophysiological mechanisms underlying PSD, which may promote the development of a theoretical basis for clinical diagnosis.

## Figures and Tables

**Table 1:** Demographic and clinical information of all participants.

	NCs (*n* = 21)	PSND (*n* = 24)	PSD (*n* = 20)	*F*/*t*/*χ*^2^	*p* value
Age^†^, mean (SD)	60.71 (10.36)	61.67 (7.21)	66.95 (9.04)	2.94	0.06
Male^‡^, *n* (%)	9 (43)	20 (83)	11 (55)	8.27	0.02^∗^
Handiness	R	R	R	N/A	N/A
ICV (L)^†^, mean (SD)	1.46 (0.14)	1.41 (0.31)	1.30 (0.26)	8.35	0.02^∗^
FD^†^, mean (SD)	0.09 (0.07)	0.11 (0.07)	0.08 (0.05)	5.16	0.08
Education level^‡^, *n* (%)				19.36	0.01^∗^
None	0	4 (17)	7 (35)		
Primary	14 (67)	6 (25)	5(25)		
Junior high school	5 (24)	8 (33)	5 (25)		
Senior high school	2 (9)	5 (21)	3 (15)		
Superior	0	1 (4)	0		
Duration of illness (day)^†^, mean (SD)	N/A	99.04 (62.14)	104.55 (69.69)	-0.28	0.78
NIHSS^1†^, mean (SD)	N/A	1.79 (2.36)	1.55 (2.21)	0.35	0.73
NIHSS^2†^, mean (SD)	N/A	0.17 (0.38)	0.25 (0.44)	-0.67	0.51
mRS^1‡^, *n* (%)				3.03	0.39
0-1-2-4	N/A	15 (63)-7 (29)-0-2 (8)	14 (70)-5 (25)-1 (5)-0		
mRS^2‡^, *n* (%)				0.11	0.74
0-1	N/A	19 (79)-5 (21)	15 (75)-5 (25)		
MMSE^1†^, mean (SD)	25.88 (3.04)	26.88 (2.91)	20.30 (4.50)	20.77	<0.001^∗∗∗^
MMSE^2†^, mean(SD)	25.88 (3.04)	27.17 (2.32)	20.45 (4.38)	24.19	<0.001^∗∗∗^
Mini-Cog^1‡^, *n* (%)				79.46	<0.001^∗∗∗^
0-1-2-3-4-5-6-7	0-0-0-0-1 (5)-5 (24)-3 (14)-12 (57)	0-0-3 (13)-12 (50)-0-9 (37)-0-0	2 (10)-0-7 (35)-9 (45)-2 (10)-0-0-0		
Mini-Cog^1‡^, *n* (%)				65.34	<0.001^∗∗∗^
0-1-2-3-4-5-6-7	0-0-0-0-1 (5)-5 (24)-3 (14)-12 (57)	0-0-2 (8)-8 (33)-1 (5)-13 (54)-0-0	3 (15)-1 (5)-4 (20)-7 (35)-1 (5)-4 (20)-0-0		

^1^Acute phase; ^2^third month; ^†^one-way ANOVA analysis/two-sample *t*-test; ^‡^chi-square test. Age, ICV, duration of illness, and MMSE are shown as mean (standard deviation); other data (*n* (%)) are number of participants (percentage). ∗, ∗∗, and ∗∗∗ represent *p* < 0.05, *p* < 0.01, and *p* < 0.001, respectively. NCs: normal controls; PSND: poststroke nondemented; PSD: poststroke demented; ICV: intracranial volume; FD: framewise displacement; NIHSS: National Institutes of Health Stroke Scale; mRS: modified Rankin Scale; MMSE: Mini-Mental State Examination.

**Table 2:** Lesion information of all patients.

Lesion	PSND (*n* = 24)	PSD (*n* = 20)	*t*/*χ*^2^	*p* value
Location of stroke^‡^			0.45	0.80
Left	11	11		
Right	11	8		
Bilateral	2	1		
Lesion volume (ml)^†^, mean (SD)	1.28 (1.32)	0.96 (1.12)	0.87	0.39
Lesion number^‡^				
1-2-3	17 : 7 : 0	16 : 3 : 1	2.29	0.32

^†^Two-sample *t*-test; ^‡^chi-square test. PSND: poststroke nondemented; PSD: poststroke demented; SD: standard deviation.

**Table 3:** The information of stroke lesions in patients.

Patient ID	Hemisphere	Location	Number	Volume (ml)
PSND_01	Bilateral	White matter	2	0.65
PSND_02	Right	White matter, cerebellum	1	0.35
PSND_03	Left	White matter, caudate	1	2.73
PSND_04	Right	White matter	1	1.19
PSND_05	Right	White matter	1	0.36
PSND_06	Right	White matter	1	2.23
PSND_07	Left	Cerebellum	2	0.47
PSND_08	Left	Occipital lobe	1	3.22
PSND_09	Left	White matter, cingulate gyrus	2	0.52
PSND_10	Right	White matter	1	1.06
PSND_11	Bilateral	White matter	2	1.65
PSND_12	Right	Insula	1	0.21
PSND_13	Left	Occipital, temporal lobes	2	2.06
PSND_14	Left	White matter	2	0.83
PSND_15	Left	Cerebellum	1	0.12
PSND_16	Right	Thalamus	1	0.65
PSND_17	Left	Thalamus	1	0.26
PSND_18	Right	White matter	1	0.96
PSND_19	Right	White matter	1	1.90
PSND_20	Left	Cerebellum	1	0.44
PSND_21	Right	White matter	1	1.86
PSND_22	Right	White matter, frontal lobe	2	0.57
PSND_23	Left	Occipital lobe	1	6.02
PSND_24	Left	White matter	1	0.47
PSD_01	Left	White matter, frontal lobe	3	0.42
PSD_02	Left	White matter	1	0.24
PSD_03	Right	White matter, putamen, thalamus, insula	1	4.79
PSD_04	Left	White matter	1	0.28
PSD_05	Left	White matter, caudate	1	1.84
PSD_06	Left	White matter	1	0.59
PSD_07	Right	White matter	1	1.28
PSD_08	Left	White matter, putamen	2	2.27
PSD_09	Left	Thalamus	1	0.84
PSD_10	Left	White matter	1	0.35
PSD_11	Left	White matter, frontal lobe	2	0.81
PSD_12	Bilateral	White matter, putamen	2	1.43
PSD_13	Right	White matter, parietal/frontal lobe	1	2.03
PSD_14	Right	White matter	1	0.23
PSD_15	Left	Thalamus	1	0.15
PSD_16	Right	Temporal lobe	1	0.32
PSD_17	Right	Thalamus	1	0.03
PSD_18	Right	White matter, putamen	1	0.40
PSD_19	Left	White matter	1	0.34
PSD_20	Right	White matter	1	0.48

**Table 4:** Brain regions showing significant differences in GMV and fALFF values among the PSND, PSD, and NC groups.

Region	Hemisphere	MNI coordinate			Cluster size	*F* value
		*X*	*Y*	*Z*		
GMV						
Cerebellum posterior lobe	Left	-12	-64.5	-36	417	20.85
Medial prefrontal cortex	Left	-3	70.5	-9	711	24.57
Superior frontal gyrus	Left	-22.5	28.5	60	511	16.01
Superior temporal gyrus	Right	60	-24	21	633	14.91
fALFF						
Inferior frontal gyrus	Right	45	3	48	138	11.16

GMV: gray matter volume; fALFF: fractional amplitude of low-frequency fluctuation; NC: normal control; PSND: poststroke nondemented; PSD: poststroke demented.

**Table 5:** Results of ROC curve analysis for classification of PSD and PSND patients.

Variables	AUC	SE	*p* value	CI
CPL_L	0.804	0.066	0.001	0.675-0.933
mPFC_L	0.531	0.093	0.724	0.350-0.713
SFG_L	0.502	0.092	0.981	0.322-0.682
STG_R	0.783	0.071	0.001	0.644-0.923
IFG_R	0.717	0.077	0.014	0.565-0.868

AUC: area under the curve; SE: standard error; CI: confidence interval; CPL_L: left cerebellum posterior lobe; mPFC_L: left medial prefrontal cortex; SFG_L: left superior frontal gyrus; STG_R: right superior temporal gyrus; IFG_R: right inferior frontal gyrus.

## Data Availability

The data that support the findings of this study are available from the corresponding author upon request.
